# Regulation of angiogenesis through the efficient delivery of microRNAs into endothelial cells using polyamine-coated carbon nanotubes

**DOI:** 10.1016/j.nano.2016.02.017

**Published:** 2016-08

**Authors:** Andrea Masotti, Mark R. Miller, Antonella Celluzzi, Lorraine Rose, Federico Micciulla, Patrick W.F. Hadoke, Stefano Bellucci, Andrea Caporali

**Affiliations:** a Bambino Gesù Children's Hospital-IRCCS, Gene Expression-Microarrays Laboratory, Rome, Italy; b University/British Heart Foundation Centre for Cardiovascular Science, The Queen's Medical Research Institute, University of Edinburgh, Edinburgh, UK; c INFN-Laboratori Nazionali di Frascati, Rome, Italy

**Keywords:** MicroRNA, Endothelial cell, Carbon nanotube, Polyamine, Angiogenesis

## Abstract

MicroRNAs (miRNAs) directly regulate gene expression at a post-transcriptional level and represent an attractive therapeutic target for a wide range of diseases. Here, we report a novel strategy for delivering miRNAs to endothelial cells (ECs) to regulate angiogenesis, using polymer functionalized carbon nanotubes (CNTs). CNTs were coated with two different polymers, polyethyleneimine (PEI) or polyamidoamine dendrimer (PAMAM), followed by conjugation of miR-503 oligonucleotides as recognized regulators of angiogenesis. We demonstrated a reduced toxicity for both polymer-coated CNTs, compared with pristine CNTs or polymers alone. Moreover, polymer-coated CNT stabilized miR-503 oligonucleotides and allowed their efficient delivery to ECs. The functionality of PAMAM-CNT-miR-503 complexes was further demonstrated in ECs through regulation of target genes, cell proliferation and angiogenic sprouting and in a mouse model of angiogenesis. This comprehensive series of experiments demonstrates that the use of polyamine-functionalized CNTs to deliver miRNAs is a novel and effective means to regulate angiogenesis.

## Graphical abstract

CNTs were functionalized with polyethyleneimine (PEI) or polyamidoamine (PAMAM) polymers to develop delivery systems for effectively transferring miRNA oligonucleotides into endothelial cells (ECs). We have shown a lower cell toxicity of polymer-functionalized CNTs compared to pure polymers. Most importantly, we have demonstrated that CNTs can mediate a stable intracellular delivery of miRNA oligonucleotides to ECs, thus regulating target gene expression and EC functions *in vitro* and *in vivo*.

**Figure:**
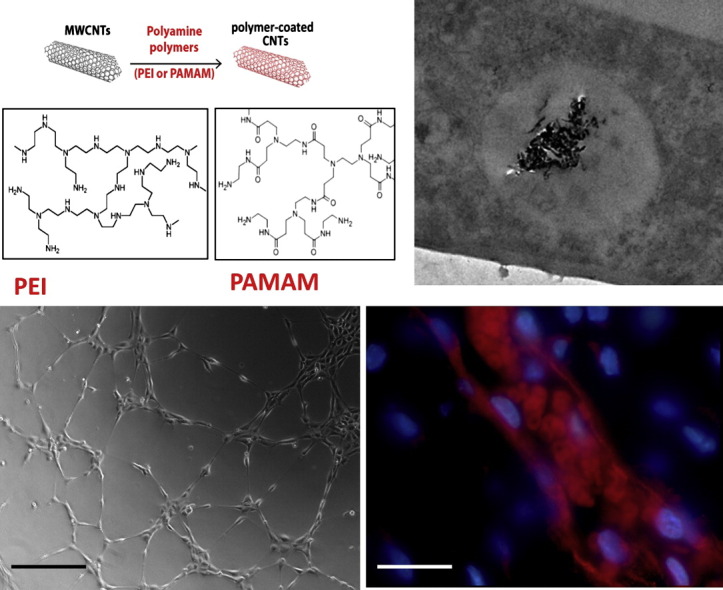

MicroRNAs (miRNAs) are a class of non-coding RNAs that function as critical regulatory elements targeting multiple functionally-related genes. The significant role of miRNAs in developmental biology and in diseases such as cancer, diabetes and cardiovascular disease has recently attracted considerable research interest.^1^, ^2^ Recent studies have revealed important roles for miRNAs in regulating angiogenesis, particularly via regulation of endothelial cell (EC) function.^3^

The term angiogenesis is used to generally indicate the growth and remodeling process of the primitive vascular network into a complex network during pre-natal development. After birth, reparative angiogenesis is activated during wound healing and in response to vascular injury, while pathological angiogenesis contributes to tumor growth and pulmonary hypertension. While it is desirable to block the growth of new blood vessels under these circumstances, the controlled stimulation of angiogenesis is beneficial when the local blood supply is impaired.^4^

Since adaptation and remodeling of vasculature also involve epigenetic components such as microRNAs, the modulation of their expression through innovative technologies would represent a way to regulate the expression of multiple genes, thus opening new avenues for vascular therapeutics. Regulation of member of miR-16 family, including miR-503, controls EC function and angiogenesis in different murine models of vascular disease (reviewed in^5^). We have previously shown that local inhibition of miR-503 restores post-ischaemic angiogenesis in diabetic mice.^6^ Finally, miR-503 is also involved in regulation of EC proliferation during development of pulmonary hypertension. Lung delivery of miR-503 mimics inhibits pulmonary artery EC proliferation and could alleviate PAH pathogenesis.^7^

Although miRNA-based therapeutic interventions are being considered, they are currently underdeveloped for a range of clinical cardiovascular applications.^8^

The major challenge of miRNA-based therapies is an existing need to increase the delivery and stability of miRNA regulators, while minimising off-target effects.^9^ Naked miRNA mimics or anti-miRNAs are highly charged and often unstable in the circulation; thus development of suitable carriers represents an attractive means to target delivery to diseased areas.^10^ Moreover, cell interaction in the presence of serum proteins is a limiting factor for many nanotechnology approaches to RNA delivery.^11^

Several strategies have been reported for the delivery of miRNAs, including liposomes,^12^ biodegradable chitosans^13^ and adeno-associated virus or lentivirus.^14^ In recent years, much effort has been made in developing non-viral vector systems for gene therapy.^11^ These systems could provide better biocompatibility and lower toxicity in comparison with the viral carriers, as well as development for a large-scale production. The advent of nanotechnology (development of materials with at least one dimension of < 100 nm) has the potential to revolutionize many forms of industry, and offers novel possibilities for biomedical applications and drug delivery.^15^ A variety of nanoparticles constructed from lipids, polymers and metals have already been evaluated as delivery systems for siRNAs,^16^ and similar conceptual frameworks have been applied to miRNA mimics and anti-miRNAs.^17^, ^18^

Carbon nanotubes (CNTs) have recently gained high popularity as potential drug carriers, therapeutic agents and diagnostic tools.^19^ CNTs are one dimensional cylindrical graphene sheets, either as a single-wall (single wall carbon nanotube; SWNTs) or multiple coaxial walls (multi-walled carbon nanotubes; MWNTs). Due to their hydrophobic surfaces, unmodified (‘pristine’) CNTs are not soluble in aqueous solutions and readily agglomerate into large particle clusters. Surface functionalization is required to suspend CNTs in a way that minimises agglomeration and renders them biocompatible for their medical applications.^20^ Furthermore, use of cationic molecules or polymers, such as polyethyleneimine (PEI) or polyamidoamine dendrimer (PAMAM), to improve CNTs functional properties leads to their electrostatic interaction with the negatively charged siRNAs or plasmid DNA, thus increasing nucleic acid loading on nanomaterials.^21^, ^22^ Finally, functionalization of CNTs may also facilitate their entry into the cells by endocytosis or by penetrating directly through cell membranes, thus transporting molecules of interest across the cytoplasmic and nuclear membranes without toxicity.^23^, ^24^ Nano-formulations based on functionalized CNTs have successfully been used to deliver siRNA in *in vitro* and *in vivo* models.^25^

In this study, pristine CNTs were functionalized with a high molecular weight branched PEI or PAMAM to develop two effective delivery systems for transferring miR-503 oligonucleotides into ECs. We demonstrate that ECs can rapidly internalize the miR-503-carrying CNTs and that polymer-coated CNTs exhibit lower toxicity than the pristine CNTs or pure polymers. In particular, CNTs coated with PAMAM show better transfection efficiency compared with the PEI-CNT formulation. Most importantly, we demonstrate that PAMAM-coated CNTs increase the stability of miR-503 oligonucleotides and, consequently, regulate target gene expression and angiogenesis *in vivo*.

## Methods

A detailed version of the Methods is available as Supplementary Materials.

## Results

### Preparation of polyamine-coated CNTs

**Figure 1:**
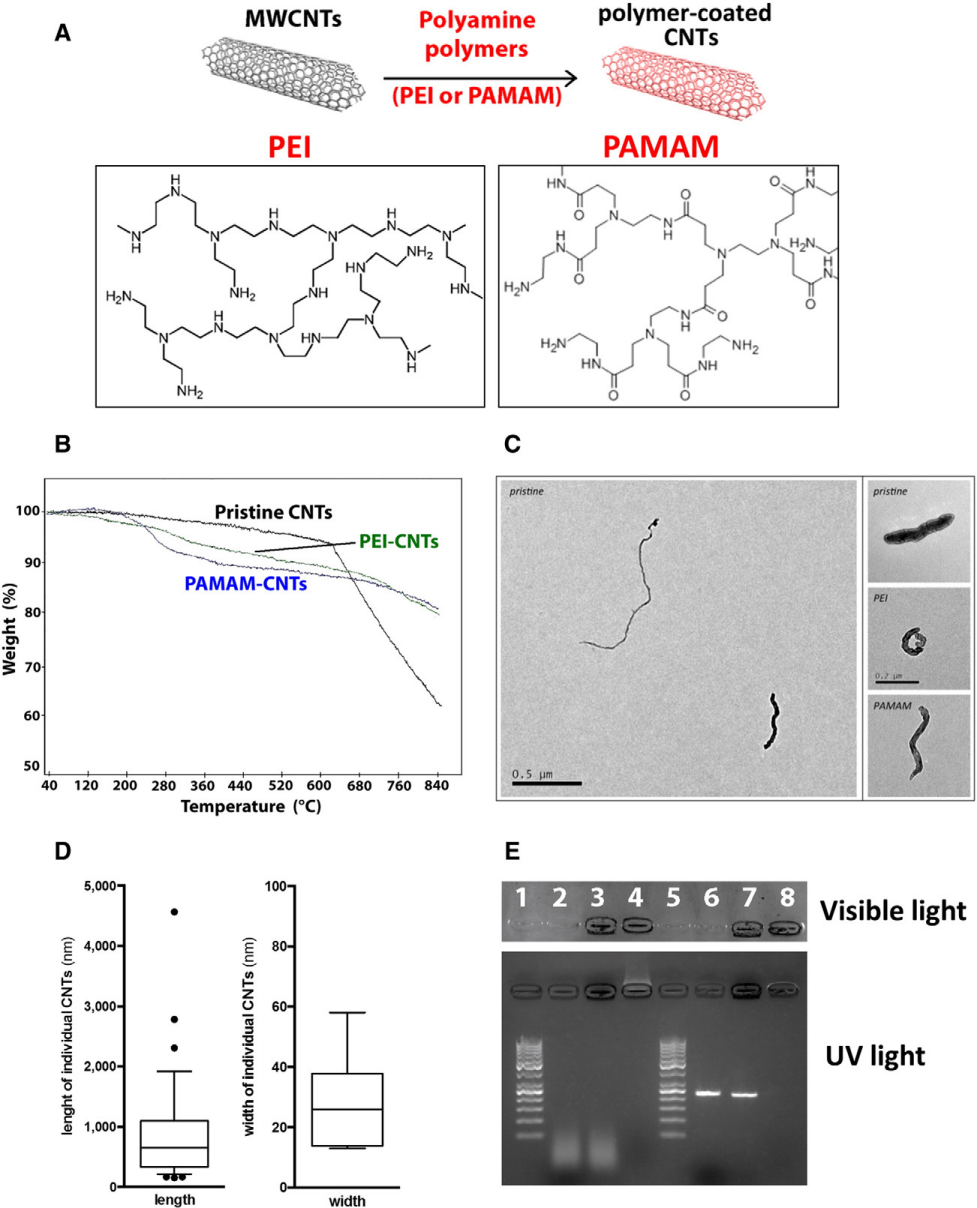
Polymer coating and dimensions of carbon nanotubes. **(A)** Schematic representation of polymer-coated (PEI and PAMAM) CNT preparation. Boxes report only a part of the PEI and PAMAM molecular structure. **(B)** Thermal analysis (TGA) performed on pristine CNTs, PEI-CNTs and PAMAM-CNTs. **(C)** Representative transmission electron microscopy (TEM) images of individual CNTs. Main panel showing inherent differences in CNT length and width, with small panels showing higher magnification of each type of CNT (scale bar = 200 nm). **(D)** Length and width of pristine CNTs derived from TEM images. Box and whisker plots with central bar indicating median value, box indicating interquartile range, and whiskers 10%-90% range (length: N = 33 from n = 5 subsamples; width: N = 6 from high resolution images of n = 3 subsamples). **(E)** Agarose gel (1%) stained with Hoechst 33258 showing that PEI-coated CNTs are able to bind DNA (5:1 w/w) (lanes 4 and 8) compared to uncoated/pristine CNTs (lanes 3 and 7). DNA ladder (50 bp) was loaded in lanes 1 and 5, fragmented DNA (< 50 bp) in lane 2 and a well-characterized DNA fragment (~ 250 bp) in lane 6.

Polyamine-coated CNTs were prepared by overnight incubation of pristine CNTs in the presence of PEI polymer (PEI-CNTs) or PAMAM dendrimer (PAMAM-CNTs) (Figure 1, A). The polymer/dendrimer adsorption onto CNTs occurs spontaneously, likely through hydrophobic interactions, although the precise physicochemical mechanism and molecular interactions still remain to be defined. To evaluate the amount of PEI or PAMAM polymer bound to CNTs, we exploited the colorimetric reaction of ninhydrin^26^ whereby free amine groups of unbound polymer can be monitored by spectrophotometry. The amount of PEI (or PAMAM) bound to CNTs was modest (4.2 ± 0.1% of the starting polymer amount for both compounds, which correspond to a final polymer/CNTs weight ratio of 1:10) but sufficient to allow further complex formation with oligonucleotides. Thermal analysis (TGA) performed on PEI-CNTs and PAMAM-CNTs (Figure 1, B) confirmed that the weight loss of these two compounds is ~ 12%-14% at a temperature where the weight loss of the pristine CNTs is approximately 4%-5%.

### Physicochemical characterization of polyamine-coated CNTs

Transmission electron microscopy (TEM) of polyamine-coated CNT suspensions did not show significant differences between the pristine and the polymer-coated CNTs (Figure 1, C). The majority of CNTs had a length between 200 nm and 1200 nm, with a median length of 652 nm (interquartile range: 332-1098 nm; N = 33; Figure 1, D). High resolution TEMs images showed two groups of CNTs which in terms of width (diameter) varied from either ~ 13-14 nm or 26-58 nm (Figure 1, D). These values are in broad agreement with the concentric layer morphology of multi-walled CNTs and the nominal diameter of the original material (20-40 nm). Dynamic light scattering did not display differences between the dimensions of individual nanotubes with or without PEI or PAMAM grafting, suggesting that the coating with these polymers did not alter the dimension of CNTs in the experimental conditions employed (data not shown). The ζ-potential of diluted suspensions of PEI-CNTs and PAMAM-CNTs indicated positively charged values (11.7 ± 1.9 and 18.9 ± 1.8 mV, respectively) and strongly suggested that these compounds can bind nucleic acids.

### Polyamine-coated CNTs are able to bind nucleic acids

To assess the ability of polyamine-coated CNTs to bind oligonucleotides, we performed agarose gel retardation assays (Figure 1, E). Polyamine-coated CNTs were able to bind efficiently both small DNA fragments (< 50 nt) (similar to miRNA-mimics) and longer fragments (~ 250 bp) by forming large polyplexes that are unable to migrate through the gel (Figure 1, E, lanes 4 and 8). Uncoated CNTs did not form complexes with DNA under these experimental conditions (Figure 1, E, lanes 3 and 7) or migrate through the gel. PAMAM-CNTs displayed similar DNA binding properties (data not shown). Thus, despite relatively low levels of polymer bound to CNTs, polyamine-coated CNTs were still able to bind DNA efficiently.

### Cytotoxicity of polyamine-coated CNTs

**Figure 2:**
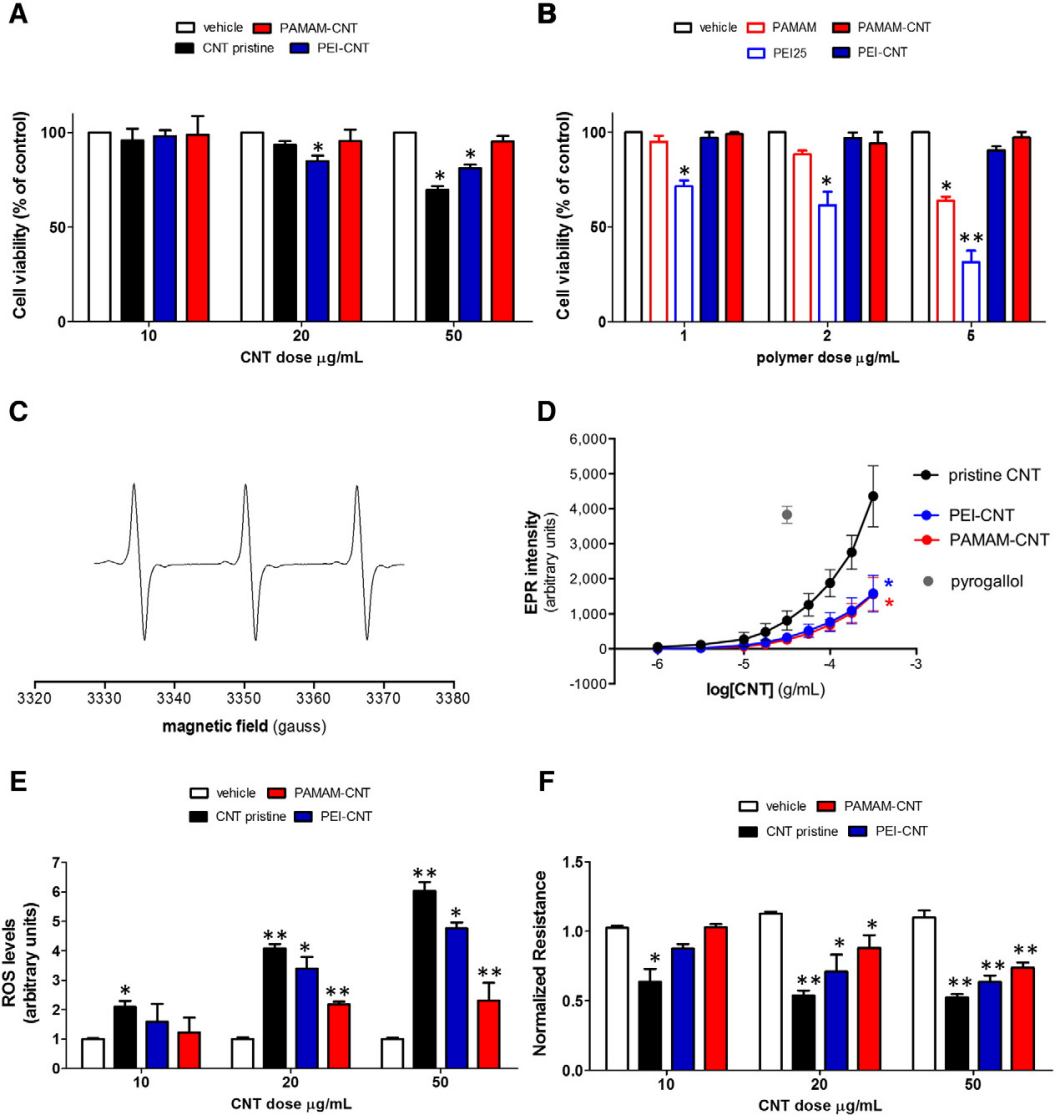
*In vitro* evaluation of toxicity, ROS production and EC permeability by polyamine-CNTs. Cell viability of **(A)** PEI and PAMAM-coated CNTs or **(B)** in comparison with unbound PEI and PAMAM was determined by MTT assay and expressed as the percentage of the optical density at 570 nm of treated cells relative to vehicle. Mean ± SEM (n = 5), *P < 0.05, **P < 0.01 vs NT. **(C)** Representative electron paramagnetic resonance (EPR) spectra from reaction of Tempone-H with superoxide generated from pristine CNTs. The amplitude of peaks indicates the magnitude of free radical production. **(D)** Superoxide free radical generation from CNTs in the absence of cells, quantified by EPR. Pristine CNTs (black) generated significantly more superoxide than PEI-coated CNTs (blue) or PAMAM-coated CNTs (red). Pyrogallol (grey; 30 μmol/L) was used as positive control for generation of superoxide. Mean ± SEM (n = 5 for CNTs, n = 7 for pyrogallol). *P < 0.05 compared to pristine CNT, one-way ANOVA with Tukey's post-hoc test. **(E)** Total ROS production from HUVECs treated with CNTs. Equivalent DMSO-treated cells have been used as control. Mean ± SEM (n = 5), *P < 0.05 or **P < 0.01 vs DMSO. **(F)** Effect of CNTs on the permeability of the endothelial monolayer evaluated using an electric cell substrate impedance sensing (ECIS) system. Mean ± SEM (n = 3), *P < 0.05 or **P < 0.01 vs untreated cells.

Human umbilical vein endothelial cells (HUVECs) were treated with PEI-CNTs or PAMAM-CNTs (10-50 μg/mL) and cytotoxicity measurements are given in Figure 2, A. Viability of HUVECs decreased with increasing concentrations of PEI-CNTs or PAMAM-CNTs, with low levels of cytotoxicity occurring at concentrations above 20 μg/mL (Figure 2, A). Interestingly, at the highest concentration tested, both polyamine-coated CNTs showed reduced cell toxicity compared with pristine CNTs (Figure 2, A). In particular, PAMAM-CNTs exhibited a lower toxicity compared with PEI-CNTs or pristine CNTs. Cell toxicity of polymer coated-CNTs was also compared with the equivalent concentration of unbound polymer (i.e. without CNTs). Surprisingly, unbound polymers were relatively toxic at low concentrations (1 μg/mL; equivalent concentration to 10 μg/mL polyamine-bound CNTs). At a dose of 5 μg/ml (equivalent to 50 μg/mL polyamine-CNTs), less than 20% or 60% of cells were viable in the presence of PEI or PAMAM respectively, while 70% or 90% of cells were viable when incubated with PEI-CNTs or PAMAM-CNTs, respectively (Figure 2, B). As reported in other studies in different cell lines,^22^, ^27^ these results show that polymers coated with CNTs were less cytotoxic to HUVECs cells compared to polymer only or pristine CNTs.

### Free radical generation by pristine and polyamine-coated CNTs

Generation of cellular oxidative stress through free radical generation is a consistently proposed mechanism for the toxicity of nanoparticles.^28^ Incubation of pristine or polymer-coated CNTs indicated that pristine or polyamine-coated nanotubes generated EPR-detectable free radicals in physiological buffer (Figure 2, C). Free radical production after 60 min incubation was linear over time (data not shown), and significantly increased as a function of the concentration of nanotubes (Figure 2, D). Pristine CNTs generated significantly more free radicals than either the PEI- or PAMAM-CNTs indicating that polymer-coating has a favorable anti-radical effect. The assay confirmed that pristine CNTs were able to induce significant levels of reactive oxygen species (ROS) production in HUVECs in a dose-dependent manner. As with innate CNT-derived free radicals, polymer-coated CNTs produced a lower level of ROS, especially PAMAM-coated CNTs (Figure 2, E).

### Regulation of endothelial permeability by CNTs

The alteration of vascular permeability has been suggested to have a crucial role not only in the pathogenesis of several diseases (i.e. cardiovascular diseases, diabetes, insulin resistance, chronic kidney failure, and tumors),^29^ but also on drug delivery to cells, tissues, and organs.^30^ We then investigated whether exposure of ECs to nanotubes led to changes in EC barrier function using electric cell substrate impedance sensing (ECIS).^31^ ECs treated with pristine CNTs displayed a significant decrease in impedance compared with control cells, indicating an increase in cell monolayer permeability (Figure 2, F). Significant changes were seen at the lowest concentration (10 μg/mL) of pristine CNTs, whereas polymer-coated CNTs increased cell monolayer permeability only at the high dose.

### Delivery of miRNAs in endothelial cells by polyamine-coated CNTs

**Figure 3:**
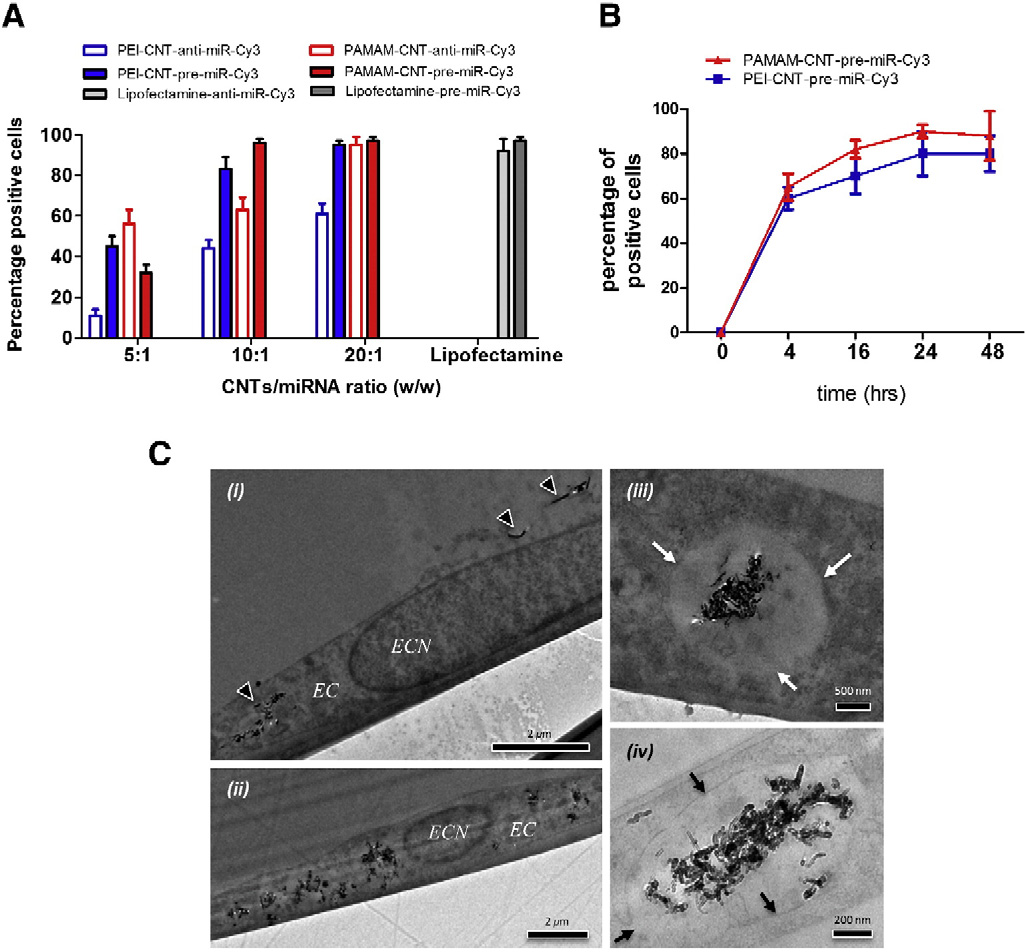
Polyamine-coated CNTs deliver miRNA to endothelial cells. PEI-CNTs and PAMAM-CNTs (10-50 μg/mL) were complexed with pre- or anti-miRNA (50 nM) Cy3-labelled oligonucleotides and incubated with HUVECs. **(A)** Flow cytometry analysis of HUVECs treated with CNTs complexed with pre- or anti-miRNA or transfected with Lipofectamine. Efficiency of transfection is expressed as percentage of Cy3-positive cells. Mean ± SEM (n = 5). **(B)** Time course of the uptake of CNT-miR-Cy3 in HUVECs. Mean ± SEM (n = 5) **(C)** Representative transmission electron microscopy images showing penetration of PAMAM-CNTs/pre-miRNA (10:1 w/w) into endothelial cells at 48 h. *EC* = endothelial cell, *ECN* = endothelial cell nucleus. Arrow heads in *(i)* show carbon nanotubes. Arrows in *(iii)* & *(iv)* show membrane of vacuole surrounding CNTs.

In order to verify the delivery efficiency of polymer-coated CNTs to HUVECs, PEI-CNTs and PAMAM-CNTs were complexed with Cy3-labelled oligonucleotides as a quantifiable surrogate for miRNA precursor (pre-miRNA) or miRNA inhibitor (anti-miRNA) and quantified by flow cytometry (FACS) after 24 h (Figure 3, A). At 5:1 weight ratio polyamine-CNTs/pre-miRNA or polyamine-CNTs/anti-miRNA, 51% vs 32% of HUVECs were labelled with PEI-CNTs and PAMAM-CNTs, respectively. At 10:1 weight ratio 89% of HUVECs were labelled with PEI-CNTs, whereas PAMAM coating increased the transfection efficacy to 99%. PAMAM-coated CNTs were able to deliver anti-miR more efficiently than PEI-coated CNTs (Figure 3, A).

We have then investigated the kinetic of the uptake of Cy3-labelled oligonucleotides complexed with CNTs in HUVECs. We used PAMAM or PEI CNT/pre-miR complex (10:1 w/w) and we followed the cellular uptake by FACS for 48 h. Already after 4 hr, labelled-CNTs are detected inside the 60% of the cells, while at 24 hr the majority of the cells displayed the Cy3-labelled CNTs (Figure 3, B).

To confirm the intracellular localization of CNTs/pre-miRNA complex, we observed HUVECs by transmission electron microscopy (TEM) (Figure 3, C). TEM images did not show any visible evidence of cytotoxicity or cell activation in HUVECs treated with CNTs/pre-miRNA (w/w 10:1). Following 24 h incubation of HUVECs, CNTs were visible adhering to the cell surface and, importantly, inside cells (Figure 3, C (i)). The CNTs were observed in the cell cytoplasm but not within cell nuclei. In some cases the CNTs appeared to be distributed freely in cell cytoplasm (Figure 3, C (ii)), whereas often they were visible as aggregates within vacuoles (Figure 3, C (iii) & (iv)).

### Functional miR-503 is delivered to endothelial cells by polymer-coated CNTs

**Figure 4:**
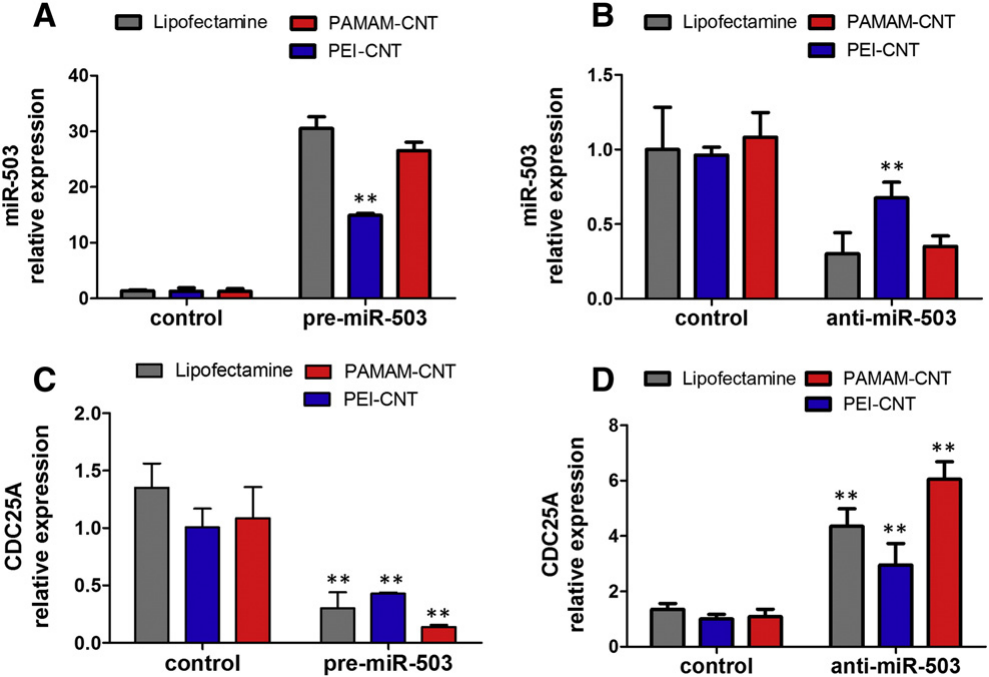
Polyamide CNTs efficiently deliver miR-503 oligonucleotides. Expression of miR-503 in HUVECs treated with PEI-CNTs or PAMAM-CNTs conjugated with **(A)** pre-miR or **(B)** anti-miR-503 oligonucleotides (10:1 w/w). The commercially available transfection agent Lipofectamine was used as positive control, and miRNA scramble sequence as negative controls. Expression was normalized to snRU6. Mean ± SEM (n = 5). **P < 0.01 vs Lipofectamine. Efficiency of delivery assessed by measurement of expression of *CDC25A* following **(C)** pre-miR-503 or **(D)** anti-miR-503 bound to CNTs. Expression was normalized to 18S. Mean ± SEM (n = 5). *P < 0.05 or **P < 0.01 vs control.

qPCR of miR-503 expression was used to confirm that polyamine-coated CNTs can be used as efficient miRNA delivery systems, using both anti- and pre-miR-503 oligonucleotide-bound CNTs, in comparison with that of the commercial transfection agent RNAiMAX Lipofectamine. Both polyamine-CNTs transferred anti- or pre-miR-503 oligonucleotides to the recipient cells, as demonstrated by the regulation of levels of miR-503 detected in HUVECs. PAMAM-CNTs showed miRNA oligonucleotide delivery efficiency similar to the commercial transfection agent (Figure 4, A and B). We have previously demonstrated that over-expression of miR-503 *in vitro* and *in vivo* considerably decreases cell proliferation target *CDC25A*.^6^ Loaded-CNTs regulated *CDC25A* mRNA levels in accordance with their ability to change miR-503 expression (Figure 4, C and D), whereas this effect was not observed in cell transfected with scramble sequence miRNA (control groups). Consistent with a better efficiency of transfection shown by PAMAM-CNTs (Figure 4, A), these dendrimer-coated CNTs are more effective in regulating *CDC25A* expression than PEI-CNTs. Overall, our results show that polymer-coated CNTs allow the translocation of miR-503 into HUVECs and that miRNA is efficiently released from CNT-complexes and remains functionally active.

### *In vitro* activity of CNT-miR-503 formulations in endothelial cells

**Figure 5:**
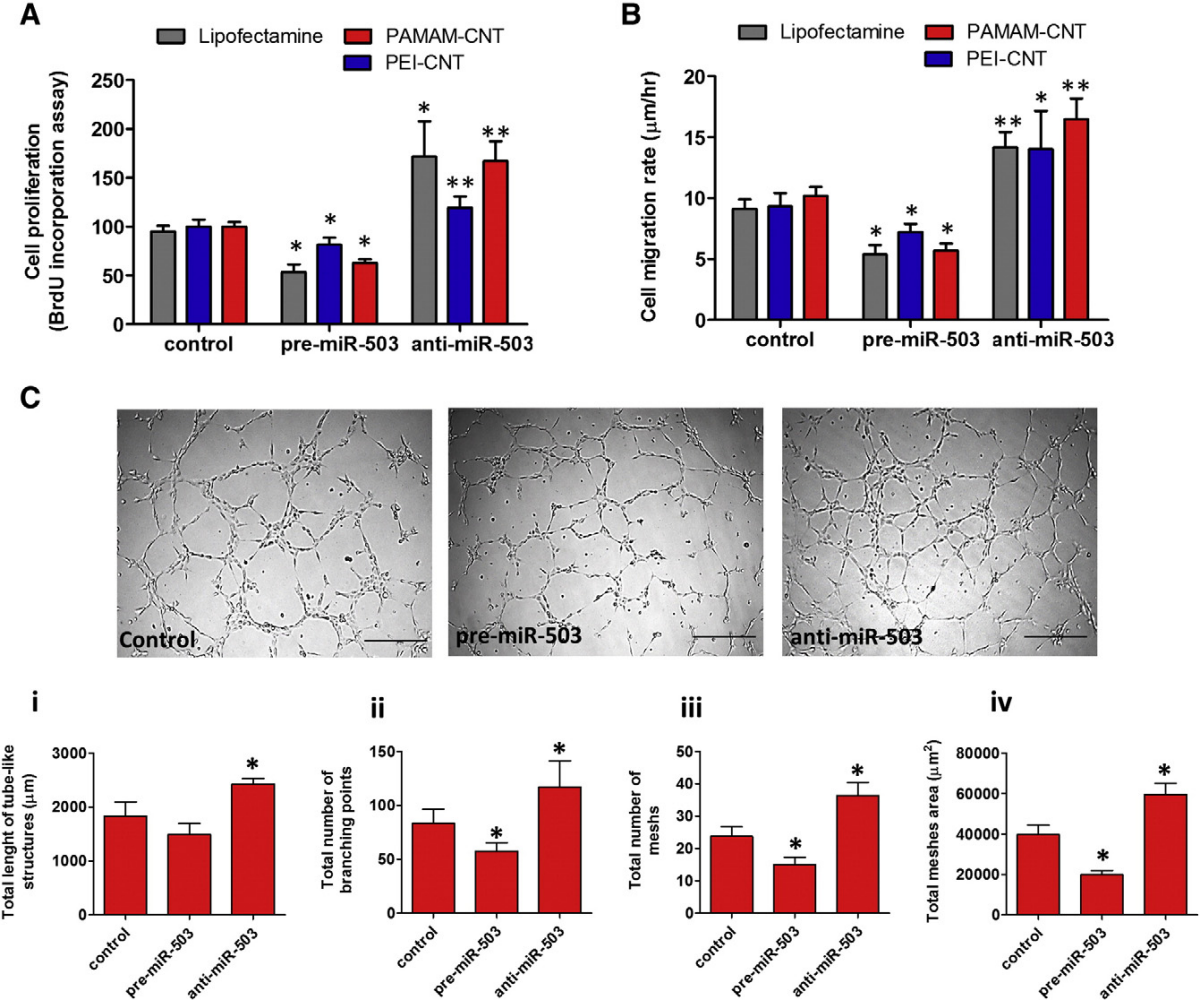
Delivery of miR-503 by CNTs leads to functional changes in endothelial cell proliferation and tube formation. Effect of CNTs conjugated with pre-miR or anti-miR-503 oligonucleotides (10:1 w/w) on **(A)** proliferation (BrdU incorporation assay) and **(B)** migration of HUVECs. Mean ± SEM (n = 5). *P < 0.05 or **P < 0.01 vs control (scramble sequence oligonucleotides). **(C)** Effect of pre- or anti-miR-503 delivery by PAMAM-CNTs on HUVECs to assess their capacity to induce formation of vascular networks. Top panel: representative images of tube-like structure of HUVECs on Matrigel. (Magnification 100 ×; scale bar 100 μm). Bottom panel: quantification of **i)** total length of the tube-like structures, **ii)** total number of junctions, **iii)** total number of meshes and **iv)** total meshes area by ImageJ software. Mean ± SEM (n = 5). *P < 0.05 or **P < 0.01 vs negative control.

The biological consequences of miR-503 oligonucleotides delivered by polyamide-coated CNTs were assessed by monitoring EC proliferation using BrdU and EC migration. Pre-miR-503 strongly decreased HUVEC proliferation, whereas anti-miR-503 exerted the opposite effect. In agreement with the level of expression of *CDC25A*, PAMAM-CNTs show a stronger effect on HUVEC proliferation than PEI-CNTs (Figure 5, A). In addition, we have demonstrated that polymer-coated CNTs conjugated with pre-miR-503 also decrease EC migration rate while conjugation with anti-miR-503 increases EC capacity to migrate (Figure 5, B).

To assess the regulation of the ability to form connections by anti-miR-503/pre-miR-503 complexed with PAMAM-CNTs, HUVECs were cultured on Matrigel. As expected, cells transfected with anti-miR-503 yielded vascular networks with a greater tube length (Figure 5, C i), more branching points (Figure 5, C ii), and an increased number of meshes (Figure 5, C iii) and meshes area (Figure 5, C iv) compared to HUVECs transfected with scrambled sequence miRNA. On the contrary, HUVECs transfected with pre-miR-503 reduced the majority of these parameters compared to controls (Figure 5, C).

### PAMAM-CNT-miR-503 complex increases miR-503 stability and angiogenesis *in vivo*

**Figure 6:**
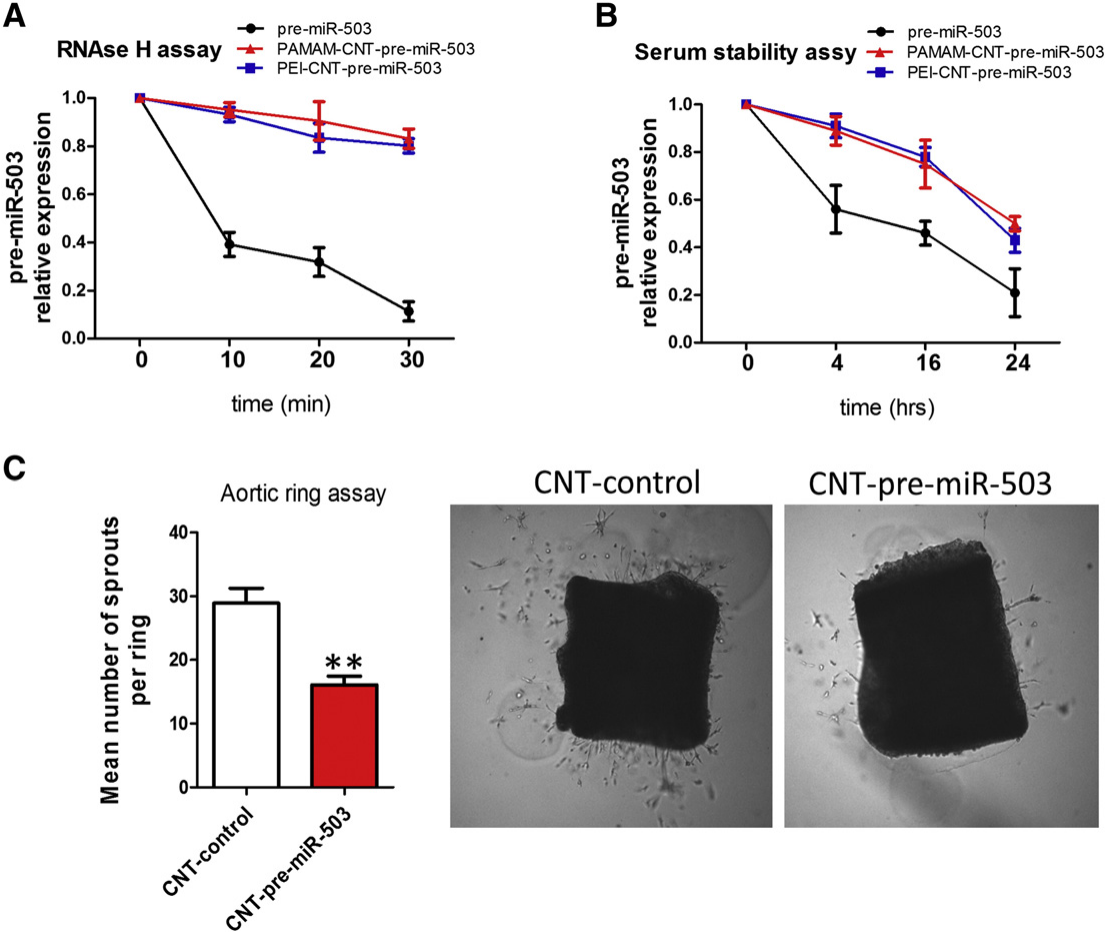
Stability of PAMAM-CNT-miR-503 and effect on *ex vivo* model of angiogenesis. **(A)** Kinetics of RNAse H digestion. Values are expressed as pre-miR-503 expression compared to pre-miR-503 values at time 0. Mean ± SEM (n = 6). **(B)** Stability of pre-miR-503 conjugated with polyamine-CNTs in serum. Values are expressed as pre-miR-503 expression compared to pre-miR-503 values at time 0. Mean ± SEM (n = 6). **(C)** Influence of PANAM-CNT-pre-miR-503 on vessel outgrowth from mouse aortic rings. **P < 0.01 vs CNT-control. Mean ± SEM (n = 6/group) and representative microscopy images of vessels outgrowth from mouse aortic ring in the presence of PAMAM-CNT-control or PAMAM-CNT-pre-miR-503.

The delivery of unmodified RNA oligonucleotides *in vivo* is greatly limited by their sensitivity to nucleolytic degradation in serum. Therefore, the stability of polyamine-coated CNTs/miR-503 complexes to RNAse H or serum degradation was analysed further. As shown in Figure 6, A, pre-miR-503 oligonucleotides conjugated with polyamine-CNTs were resistant to RNAse H degradation. Similar results have been obtained when polyamine-CNTs/pre-miR-503 complexes were incubated in serum up to 24 h (Figure 6, B). However, the unconjugated pre-miR-503 oligonucleotides undergone degradation after 10 min in the presence of RNAse H or after 4 h in serum. These data supported the evidence that the conjugation of miRNAs with polyamine-coated CNTs significantly stabilized the oligonucleotides against nucleolytic degradation.

We next explore whether PAMAM-CNT-miR-503 formulation reduced microvessels sprouting in a three-dimensional *ex vivo* mouse aortic ring angiogenesis assays. In this model, developing microvessels undergo many key features of angiogenesis over a timescale similar to that observed *in vivo*.^32^ The numbers of microvessels formed from murine aortic rings cultured *in vitro* and scored after 5 days were reduced in the presence of PAMAM-CNT-pre-miR-503 as compared with controls (Figure 6, C).

**Figure 7:**
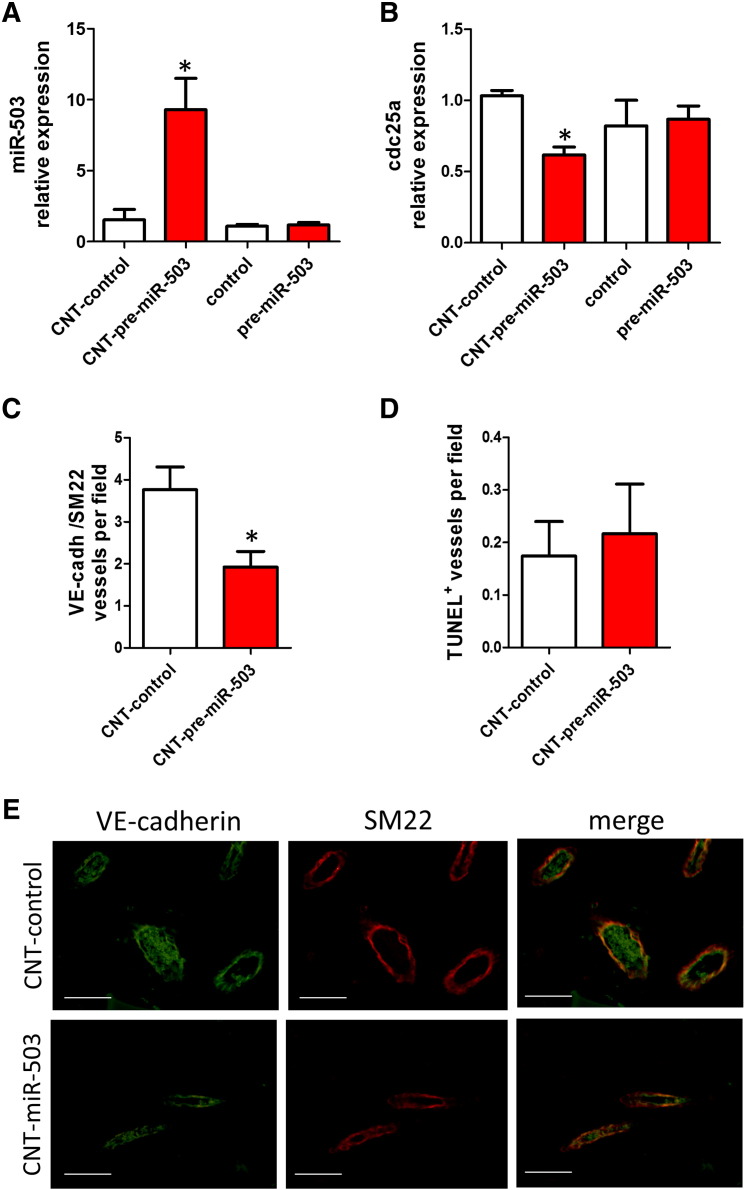
Delivery of PAMAM-CNTs-miR-503 reduces *in vivo* angiogenesis. **(A)** Expression of miR-503 or **(B)***CDC25A* in sponges treated with pre-miR-503 conjugated with PAMAM-CNTs or control oligonucleotides. The expression of miR-503 and *CDC25A* was normalized to snRU6 or 18S, respectively. Mean ± SEM (n = 6). *P < 0.05 vs CNT-control. **(C)** Quantification of VE-cadherin/SM22 positive vessels in sponges treated with PAMAM-CNT-pre-miR-503 or PAMAM-CNT-control at 21 days after implantation *P < 0.05 vs CNT-control. Mean ± SEM (n = 6/group). **(C)** Quantification of apoptosis (TUNEL assay) in vessels in the same conditions reported above. Mean ± SEM (n = 6). **(D)** Representative images of the vessels positive for VE-cadherin (green fluorescence) and SM22 (red fluorescence) in the implanted sponges (Magnification 400 ×; scale bar 100 μm).

Then, a sponge implant model was used to confirm whether the PAMAM-CNT formulation improved the stability of miRNA oligonucleotides and to study their effects on angiogenesis *in vivo*. The sponge-induced inflammatory angiogenesis consists of the implantation of a synthetic polymer in subcutaneous sites in mice.^33^ This model allows the quantitation of various components of the inflammatory angiogenic process and can be used as a controlled, sustained-release local delivery system for therapeutic agents.^34^ Pre-miR-503 oligonucleotides conjugated with PAMAM-CNTs were incorporated in the sponges and qPCR was performed using primers that can only detect exogenous miR-503. After 21 days, only the miR-503 oligonucleotides conjugated with CNTs were detected in the sponges (Figure 7, A), resulting in the silencing of its target gene *CDC25A* (Figure 7, B). Histological analysis of angiogenesis in the sponges at 21 days by quantification of VE-cadherin/SM22 vessels (Figure 7, C and E) or CD31/αSMA (Supplementary Figure 1) demonstrated that the PAMAM-CNT-pre-miR-503 formulation reduced the number of vessels compared to sponges containing only control oligonucleotides. The presence of erythrocytes in the lumen of the vessels indicated that they are linked to the host-circulation and perfused (Figure 7, E and Supplementary Figure 2). Delivery of miR-503 by PAMAM-CNTs does not increase apoptosis in the vessels (Figure 7, C), confirming that miR-503 affects *in vivo* angiogenesis through its activity on proliferation and migration of ECs as previously described.^6^

## Discussion

Here we demonstrate that coating of CNTs with PEI or PAMAM polymers is an effective means to deliver miRNAs into ECs. Importantly, these compounds exploit their transfection properties at concentrations that do not determine cytotoxic effects or loss of EC integrity. We also showed that polymer-coated CNTs conjugated with pre-miR-503 or anti-miR-503 are able to regulate cell proliferation and *in vitro* angiogenesis through the modification of their target genes. Moreover, the formulations that we have developed increase the stability of miRNA oligonucleotides in serum, paving the way for an effective use of these systems *in vivo*.

We have employed the polyamine PEI and the dendrimer PAMAM, two versatile polymers widely used in the past to obtain several drug delivery vectors for *in vitro* and *in vivo* biomedical applications.^35^, ^36^, ^37^ We have found that PAMAM-CNTs consisted in a stable dispersion of CNTs that decreased the toxicity of pristine nanotubes and limited ROS production, whereas PEI-CNTs were slightly less effective. Numerous studies have demonstrated that mammalian cells exposed to CNTs suffer from oxidative stress through generation of ROS.^38^ Indeed, CNTs in physiological buffers (in the absence of cells) generate hydroxyl and superoxide free radicals, the magnitude of which is dependent on surface area, trace metal contamination and composition of the buffer used.^39^ In the present study, we investigated both innate ROS generation and cellular oxidative stress. EPR spectroscopy was used to assess ROS generation due to the high sensitivity of this technique and because measurement is not compounded by the black colour of CNT suspensions (which generally interferes with other colorimetric assays). Furthermore, we chose the superoxide-selective spin-trap Tempone-H for the quantification of free radicals, due to the importance of the scavenging effects of superoxide on nitric oxide, a key cellular messenger in endothelial cells and cardiovascular homeostasis. Despite the well-recognized property of carbon nanotubes to generate ROS, the functionalization of CNTs with polyamine or polyamides might also impart to these nanosystems an additional antioxidant action. Given the great potential of PAMAM to limit superoxide generation from pristine nanotubes, further chemical modification of PAMAM-coated nanotubes, or improved coating efficiency, could actually inhibit the oxidative damage by ROS production that is a hallmark of cardiovascular diseases.

There are several potential routes of cellular uptake of CNTs, the extent of which will depend on many experimental parameters, such as size, length, hydrophobicity and surface chemistry.^40^ Importantly, subcellular localization of CNTs depends on the method by which CNTs enter the cell.^41^ Although the mechanism of cellular uptake is still unclear, a common pathway for the cellular uptake of functionalized CNTs is the active transport via endocytosis and inclusion of CNTs in vesicles or vacuoles.^42^ In our study, the delivery of miRNA conjugated with polymer-coated CNTs was first demonstrated by observing the fluorescence of Cy-3 labelled miRNA using flow cytometry. Interestingly, polyamine-CNTs-pre-miRNA formulations at 10:1 weight ratio are able to transfect ECs with the same efficiency of Lipofectamine. The good transfection efficiency of these systems is due to the presence of PEI and PAMAM, two polymers with excellent transfection properties.^43^, ^44^, ^45^ Another notable observation is that PAMAM-CNTs showed a higher efficacy of transfection compared with PEI-CNTs. We can speculate that the polyamide chemical structure of PAMAM can optimize complexation with miRNAs, therefore facilitating their subsequent release. We further characterized the intracellular uptake of the PAMAM-CNTs-miRNA complex. TEM images confirmed that the PAMAM-CNT-miRNA complex was indeed inside the cells' cytoplasm, and, specifically, in vacuoles.

Interestingly, the accumulation of autophagosomes in cultured human umbilical vein endothelial cells (HUVECs) treated with carboxylated multiwalled carbon nanotubes (MWCNTs) has been observed,^46^ although our systems are quite different. In any case, we cannot exclude that the hydrophilic polymer-coated CNTs employed in this study might insert spontaneously into the lipid bilayer^47^ or enter the cell by passing through the membrane as “needles”. The uptake and release of RNA-wrapped double-walled carbon nanotubes (DWNTs) by cultured human cancer cells has been studied by Neves et al who demonstrated that these nanotubes are visible within endosomes just after 30 min after transfection and are completely released by cells over a 24 h time period.^48^ In our experimental conditions, using primary EC, we have shown that CNTs are still in the cells after 48 h from transfection (Figure 3, B and C). However, we would like to highlight that different cellular systems, in particular cancer cells, have different internalization and release mechanisms. Therefore, to unravel completely the mechanisms and the kinetics of internalization and retention of our polyamine-coated CNTs and to define which process is engaged to remove them from cells, a more focused set of *in vitro* experiments are needed.

In recent years, efforts have been made to design and deliver pharmacologically-active synthetic miRNAs and, specifically, to produce oligonucleotides that bind to their miRNA targets with remarkable affinity and specificity and have suitable drug-like qualities (such as increased stability and pharmacokinetics).^8^ Our data further show that PAMAM-CNTs complexed with miR-503 precursors or inhibitors exhibited the best ratio of silencing efficacy vs toxicity in ECs, achieving strong gene expression regulation of the target gene *CDC25A* and *in vitro* angiogenesis. Following these encouraging results, PAMAM-CNT-miR-503 complex was also administered locally using a sponge implant mouse model. In our hands the sponge model is well adapted for analysis of the effects of the PAMAM-CNT-miRNAs complex on the stability and local release of miRNA oligonucleotides *in vivo* in a model of angiogenesis. We found that the PAMAM-CNT-miR-503 formulation increased the stability of miR-503 oligonucleotides, thus resulting in a prolonged silencing effect of its target gene *CDC25A* and inhibition of angiogenesis after 21 days.

Taken together our data determined that the direct conjugation of CNTs with PEI or PAMAM polymers can afford effective and stable carriers for miRNA delivery into ECs. We demonstrated that polymer-coated CNTs can release miRNAs and regulate cell proliferation and *in vitro* and *in vivo* angiogenesis through the modulation of miRNA target gene expression. In addition, our results demonstrated that the nanostructured systems developed in this work display minimal cytotoxicity, and are efficient non-viral vectors for the delivery of miRNA-based oligonucleotides to ECs, thus making them promising delivery vectors for cardiovascular applications.
