# Supplementary material for: Regulation of angiogenesis through the efficient delivery of microRNAs into endothelial cells using polyamine-coated carbon nanotubes
Source: Nanomedicine. 2016 Aug;12(6):1511–22. doi: 10.1016/j.nano.2016.02.017 (PMC4949379; doi:10.1016/j.nano.2016.02.017)
Supplement: Supplementary file 1 — Supplementary Materials and Figures. [file mmc1.docx]

**Supplementary Figure**

**
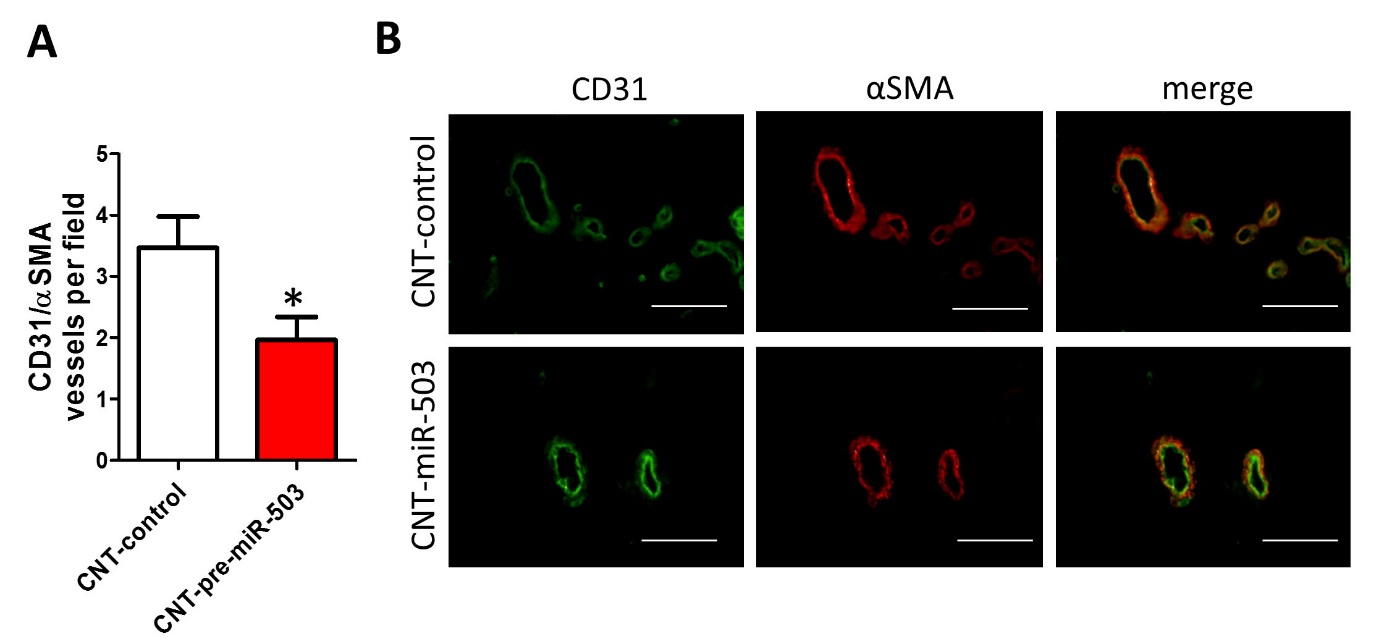
**

**Supplementary Figure 1:** (**A**) Quantification of VE-cadherin/SM22 positive vessels in sponges treated with PAMAM-CNT-pre-miR-503 or PAMAM-CNT-control at 21 days after implantation *p<0.05 vs. CNT-control. Mean ± SEM (n=6/group). (**B**) Representative images of the vessels positive for CD31 (green fluorescence) and α-SMA (red fluorescence) in the implanted sponges (Magnification 400x; scale bar 100µm).

**
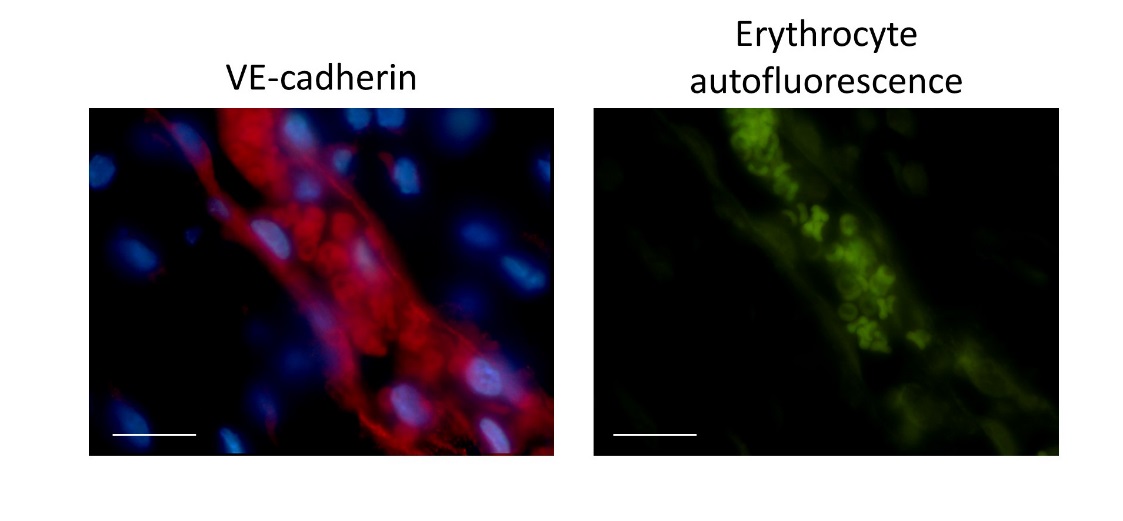
**

**Supplementary Figure 2:** Representative images of the vessels positive for VE-cadherin (red fluorescence) in the implanted sponges. Erythrocytes within perfused vessels were autofluorescent in both the red and green channels and lacked nuclei by DAPI stain. (Magnification 1000x; scale bar 100µm).

**Supplementary Methods**

***Coating and characterization of CNTs***

Multi-walled carbon nanotubes were purchased by the He Ji company ltd. (Hong Kong; cat.no. M2704), polyethylenimine polymer (PEI, cat.no. [408727](http://www.sigmaaldrich.com/catalog/product/aldrich/408727?lang=it&region=IT)) and polyamidoamine dendrimer generation 5 (PAMAM G=5, cat.no. 536709) were purchased from Sigma-Aldrich and used as received. For coating, MWCNTs (10 mg) were dispersed in distilled water (1.9 mL) and PEI (100 µl of a 200 mg/mL stock solution) or PAMAM (500 µl of pure product) were added dropwise. Suspensions were placed in a bath sonicator for 30 min and gently stirred overnight at room temperature. Samples were centrifuged at 20,800 g for 30 min and washed with distilled water three times to remove the unbound polymers. The remaining solid was suspended in distilled water (1 mL) and sonicated for 2 min prior to further use. Spectrophotometric ninhydrin assay (cat.no. 80-2118-30, Biochrom) was performed to calculate the percentage of polymer bound to carbon nanotubes (wavelength n=570). The percentage of coating was calculated by difference after comparing absorbance values of standard solutions used as calibration points. Agarose gel retardation assays were performed following standard procedures ^1^ . Dimension of CNTs were analyzed by transmission electron microscopy (TEM) (Phillips CM120 with Gatan Orius CCD camera). Mean particle diameter in suspension was measured by dynamic light scattering (Brookhaven Instruments Corp.). Thermal measurements (TGA) were carried out by using a Perkin Elmer TGS-2 thermal analyzer on the compound (1-2 mg) in a stream of N_2_ (flow rate, 50 ml/min), heating rate 10°C/min.

***Quantification of polymer bound to carbon nanotubes***

To assess the amount of polymer (PEI or PAMAM) bound to carbon nanotubes, unbound polymer was measured following overnight incubation. Spectrophotometric ninhydrin assay consisting of mixing 90 µL of a ninhydrin reagent (cat.no. 80-2118-30, Biochrom, Cambridge UK) with 10 µL of the reaction supernatant, heating at 98°C for 4 min, before diluting to a final volume of 1 mL and measuring the absorbance at 570 nm (Cary UV-Vis; Agilent*)*. The percentage of coating was calculated by difference after comparing absorbance values of standard solutions used as calibration points.

***Gel retardation assays***

Agarose gel retardation assays were prepared following standard procedures ^1^ . Polymer-coated CNTs and DNA (~500 ng) at a CNTs/DNA weight ratio of 5:1 (equivalentt to 10 µg/mL of CNTs) were incubated at 37°C for 5 min, mixed with loading buffer, loaded into 1% agarose gel (in TBE buffer), and run for 45 min at 100 V. Agarose gel contained 1 µL of an aqueous stock solution of the intercalating dye Hoechst 33258 (10 mg/mL). DNA bands and CNTs were visualized by UV illumination and visible light, respectively.

***Assessment of CNTs dimension***

Stock suspensions of CNTs (1 mg/mL) were probe sonicated (US70; Philip Harris Scientific; Lichfield, UK) for 2 min (70% power, five cycles) to minimize nanotube aggregation, then sonicated again immediately prior to use (U50; Ultrawave, Cardiff, UK; 15 min). Suspensions (5 μL) of CNTs (10 μg/mL in distilled water) were placed on a copper grid (200 mesh formvar/carbon support film; TAAB Laboratories Equipment Ltd, Aldermaston, UK) and allowed to dry in a dust free environment. Grids were viewed by transmission electron microscopy (TEM) in a Phillips CM120 transmission electron microscope (FEI UK Ltd, Cambridge, UK) and images were taken on a Gatan Orius CCD camera (Gatan, Oxon, UK).

Mean particle diameter in suspension was measured by dynamic light scattering (Brookhaven Instruments Corp., Holtsville, USA) set to the following parameters: 37^o^C, 90^o^ angle, 10x 1 min run duration; refractive index of PM: 1.5, dust cutoff: 30.

***Elemental analysis***

Elemental analysis on CNTs and polymer-coated CNTs gave the following results: Pristine CNTs (C=99.50%, H=0.50%), PEI-CNTs (C=86.7%, H=3.5%, N=9.8%) and PAMAM-CNTs (C=79.5%, H=2.9%, N=8.2%)

***Transfection of miRNAs by CNTs***

Human Umbilical Vein ECs (HUVECs) (Lonza) were grown in EGM-2 (EBM-2 medium supplemented with growth factors) and 2% Foetal Bovine Serum (FBS) (Lonza) and used between P2 and P6. HUVECs were plated in growth medium (EGM-2 2% FCS) and 50 nM (equivalent to 2 µg) of pre-miR-503 or anti-miR-503 oligonucletides (Life Technologies; PM25720 and AM25720) were incubated with polyamine-coated CNTs at different weight ratios (CNTs/oligonucleotides 5:1, 10:1, 20:1 w/w corresponding to 10, 20 or 50 µg/mL of CNTs) for 15 min at room temperature to allow complex formation. Polyamine-CNTs–oligonucleotide complexes were incubated with the cells for 4h, washed to remove excess reagents, and culture was maintained in growth medium for 24h or 48h. Lipofectamine RNAiMAX (Invitrogen) was used as control delivery vector to transfect HUVECs following manufacturer's instructions.

***Free radical generation by CNTs***

To provide a measure of CNTs reactivity, electron paramagnetic resonance (EPR) was used to establish oxygen-centered free radical generation from CNTs by the method of Miller et al. (2009) ^2^. Samples of CNTs for EPR were suspended in 200 μL physiological saline solution (Krebs buffer, composition in mM: 118.4 NaCl, 25 NaHCO_3_, 11 glucose, 4.7 KCl, 1.2 MgSO_4_, 1.2 KH_2_PO_4_, 2.5 CaCl_2_) at concentrations of 1-320 μg/mL. Samples were incubated with the spin-trap, Tempone-H (1 mM; Enzo Life Sciences, Exeter, UK), immediately before the initial measurement. Tempone-H is a highly sensitive spin-trap that shows selectivity for superoxide, forming a stable product that can be measured by EPR ^3^. Pyrogallol (30 μM) was used as a positive control to spontaneously generate superoxide radicals in this buffer ^4^. Samples were kept at 37^o^C throughout and measurements were taken after 60 min by drawing 50 μL of sample into a capillary tube (VWR International, Lutterworth, UK) and sealing with a plug of soft sealant (Cristaseal, VWR International). An X-band EPR spectrometer (Magnettech MS-200, Berlin, Germany) was used with the following parameters: microwave frequency, 9.3-9.55 Hz; microwave power, 20 mW; modulation frequency, 100 kHz; modulation amplitude, 1500 mG; center field, 3365 G; sweep width, 50 G; sweep time, 30 s; number of passes, 1. Baseline signals (Tempone-H in buffer alone) were subtracted from that of experimental readings. Free radical generation was quantified using the first derivative of the initial peak of the spectra obtained from reaction of Tempone-H with superoxide. A ‘best-fit’ curve was drawn for each individual concentration-response curve. The area under the curve was used to obtain a single value for each individual experiment and comparison between CNTs was performed by one-way ANOVA with Tukey’s post hoc tests.

***Cellular penetration by CNTs***

Penetration of CNTs into cells was assessed by transmission electron microscopy (TEM). Cells were grown on Nunc™ Thermanox™ coverslips (Thermo Fisher Scientific, Loughborough, UK) and incubations with CNTs were carried out as described above. Cell-laden coverslips were fixed for 2h in 3% glutaraldehyde (in 0.1 M sodium cacodylate buffer, pH 7.3) and post-fixed for 45 min in 1% osmium tetroxide (in 0.1 M sodium cacodylate). The samples were then dehydrated in increasing concentrations of acetone before being embedded in Araldite**^®^** resin. Sections (1 μm thick) were cut on a Reichert OMU4 ultramicrotome (Leica Microsystems Ltd, Milton Keynes, UK), stained with toluidine blue and viewed in a light microscope to select suitable areas for investigation. Ultrathin sections (60 nm thick) were cut from selected areas, stained in uranyl acetate and lead citrate, and then viewed in a Phillips CM120 Transmission electron microscope (FEI UK Ltd, Cambridge, UK). Images were taken on a Gatan Orius CCD camera (Gatan, Oxon, UK).

***RNA extraction and quantitative real time analysis***

Total RNA was extracted using the miReasy kit (Qiagen). Quantitative PCR (qPCR) was performed to measure the miR-503 expression level reverse-transcribed by the TaqMan miRNA reverse transcription kit and human miR-503 assay (Applied Biosystems) on a Lightcycler 480 (Roche). The miR-503 expression was normalized to the U6 small nucleolar RNA (snRU6). For *CDC25A* mRNA analysis, cDNA was amplified by quantitative real-time PCR (qPCR) and normalized to 18S ribosomal RNA. Each reaction was performed in triplicate and quantification was performed by the 2^-ΔΔCt^ method ^5^.

***Detection of Oxidative Stress Markers***

HUVECs were seeded on 96-well plates, cultured until reaching confluence, treated in the conditions reported above, and then assessed for reactive oxygen species (ROS) levels by detection of H2DCFDA (Molecular Probes, Paisley, UK), a marker of total oxidative stress. ^6^. Analysis of ROS was performed using INFINITE M1000 PRO plate reader (Tecan) at wavelengths of ~492–495/517–527 nm.

***Endothelial cells functional assays***

BrdU incorporation was measured using a Cell Proliferation colorimetric assay (Roche); cytotoxicity was measured using the (3-(4,5-dimethylthiazol-2-yl)-2,5-diphenyltetrazolium bromide) (MTT) cell viability assay (Promega).

***Detection of in vitro tubulogenesis by Matrigel™ assay***

The effects of CNT-control oligonucleotides, CNT-pre-miR-503 and CNT-anti-miR-503 on tubule formation were assessed by measuring HUVEC networks grown on Matrigel™ (Corning). Matrigel™ was kept on ice throughout use. Using a 96 well plate, wells were coated with 50 μL of Matrigel™ and incubated at 37^o^C for solidification. 100 μL EGM-2 was added on top of solid Matrigel before 15x10^3^ cells were added to each well. Cells were cultured over 5 hours prior to visualization and photography using a ZEISS Axiovert 25 light microscope and the MCID Core Digital Imaging software. Images were processed using the ImageJ Angiogenesis Analyzer software. (**Gilles Carpentier**. ImageJ contribution: Angiogenesis Analyzer. [ImageJ News](http://rsb.info.nih.gov/ij/notes.html), 5 October 2012)

Parameters measured using ImageJ Angiogenesis analyzer. Colours indicate 1) segments (tubule-like structures) (yellow), 2) branches (green), 3) segment enclosed areas (circular cyan shapes) and 4) master junctions (blue dots encircled in red) represent branching points.

***ECIS assays***

Endothelial cell barrier function and migration were measured in real time using an electric cell-substrate impedance sensing (ECIS) system (Applied BioPhysics, New York , USA) ^7^. HUVECs were plated on sterile 8-chambered gold-plated electrode arrays (8W10E and 8W1E) coated with cysteine (10mM) and fibronectin (10 μg/mL) and treated with CNTs. When cells reached full confluence, electrode arrays were mounted on the ECIS system within an incubator (37°C, 5% CO_2_) and monolayer resistance was recorded for 30h at 5 min intervals.

***RNAse H assay***

The RNase H assay was performed as described in ^8^. Briefly, pre-miR-503 oligonucleotides (50 nM) were conjugated with polymer-coated CNTs (10:1 w/w) and incubated at 37°C in a RNase H buffer (10 µl), which consisted in 40 mM Tris–HCl pH 7.2, 4 mM MgCl_2_, 1 mM DTT, 150 mM NaCl in the presence of 0.4 U *E.coli* RNase H (Invitrogen). The reaction was stopped by addition of EDTA (final concentration 83 mM). pre-miR-503 oligonucleotides were isolated using miReasy kit (Qiagen) and relative expression of pre-miR-503 was quantified by qPCR (Forward primer: TGCCCTAGCAGCGGGAACAGT; Reverse primer: TACCCTGGCAGCGGAAACAA)

***Serum stability assay***

Similarly, 50 nM pre-miR-503 oligonucleotides were conjugated with polymer-coated CNTs (10:1 w/w) and incubated at 37°C in 100µl of foetal calf serum (Life Technologies). Aliquotes (10 µl each) were removed after 4, 16 and 24 h and pre-miR-503 oligonucleotides were isolated using miReasy kit (Qiagen). The relative expression of pre-miR-503 was quantified by qPCR as described above for the RNAse H assay.

***Aortic ring assay***

C57Bl6 mice were killed by asphyxiation in CO_2_ in procedures approved by the University of Edinburgh ethics committee and according to the Animals (Scientific Procedures) Act 1986 (UK Home Office). The thoracic aorta was removed, washed in serum-free DMEM medium, cleaned of periadventitial tissue, and divided into 1-mm rings. Aortic rings were embedded in 200 μL of Matrigel (BD Biosciences) and incubated at 37 °C in Opti-MEM, with 10% FCS in the presence of PAMAM-CNT-control or PAMAM-CNT-pre-miR-503. The medium was changed every 48 h. All assays were performed in triplicate. The growth of new vessels was counted at day 5 by light microscopy.

***Sponge implant model and histological analysis***

Male CD1 mice aged 10-12 weeks were used in procedures approved by the University of Edinburgh ethics committee and according to the Animals (Scientific Procedures) Act 1986 (UK Home Office). Mice were anesthetized with isoflurane and a sterilized polyurethane sponge cylinder (0.5 × 1 cm) (Caligen Foam) was implanted subcutaneously on each flank as previously reported ^9^ . Phenol-red free growth factor-reduced (gfr)-Matrigel® (BD Biosciences) was used as vehicle to retain the CNTs in sponges. miRNA oligonucleotides were conjugated with CNTs at 10:1 w/w. Twenty-one days after implantation, mice were killed by asphyxiation in CO_2_ and sponges were excised. Sponges were fixed in 4% formalin, embedded in paraffin wax and sectioning for histological staining to quantify vessel density. Sections were incubated overnight at 4°C with VE-Cadherin (1:200, Abcam) or PECAM-1 (CD31) antibody (1:100, Abcam) conjugated with Alexa 488 or Alexa 594 secondary antibody (Life Technologies) to identify ECs, and Cy3-conjugated α-smooth muscle actin (αSMA) (1:100, Sigma Aldrich) or anti-SM22α (SM22) (1:100, Abcam) conjugated with Alexa 594 secondary antibody to identify vascular smooth muscle cells. Apoptosis was quantified using (TdT) dUTP Nick-End Labeling (TUNEL) assay (DeadEnd™ Promega) in combination with VE-Cadherin staining. Fluorescence microscopy was used to visualise positive staining using high power fields (400X) and the number of vessels positive for CD31/αSMA or VE-cadherin/SM22 or VE-cadherin/Tunel^+^ per field was counted. At least 20 randomly chosen fields were evaluated by an operator blinded to treatment.

***Statistical analysis***

Continuous data are expressed as mean ± SEM of at least three independent experiments. Analyses were performed using statistical comparisons two-tailed Student’s *t*-test, one-way ANOVA followed by Bonferroni or Tukey’s *post-hoc* analyses, as appropriate (GraphPad Prism v5.0). *P*<0.05 was considered statistically significant.

**References**

1. Lane D, Prentki P and Chandler M. Use of gel retardation to analyze protein-nucleic acid interactions. *Microbiological reviews*. 1992; 56: 509-28.

2. Miller MR, Borthwick SJ, Shaw CA, et al. Direct impairment of vascular function by diesel exhaust particulate through reduced bioavailability of endothelium-derived nitric oxide induced by superoxide free radicals. *Environmental health perspectives*. 2009; 117: 611-6.

3. Dikalov S, Skatchkov M and Bassenge E. Quantification of peroxynitrite, superoxide, and peroxyl radicals by a new spin trap hydroxylamine 1-hydroxy-2,2,6,6-tetramethyl-4-oxo-piperidine. *Biochem Biophys Res Commun*. 1997; 230: 54-7.

4. Taylor EL, Rossi AG, Shaw CA, Dal Rio FP, Haslett C and Megson IL. GEA 3162 decomposes to co-generate nitric oxide and superoxide and induces apoptosis in human neutrophils via a peroxynitrite-dependent mechanism. *Br J Pharmacol*. 2004; 143: 179-85.

5. Schmittgen TD and Livak KJ. Analyzing real-time PCR data by the comparative C(T) method. *Nature protocols*. 2008; 3: 1101-8.

6. Brandt R and Keston AS. Synthesis of Diacetyldichlorofluorescin: A Stable Reagent for Fluorometric Analysis. *Analytical biochemistry*. 1965; 11: 6-9.

7. Lee JF, Zeng Q, Ozaki H, et al. Dual roles of tight junction-associated protein, zonula occludens-1, in sphingosine 1-phosphate-mediated endothelial chemotaxis and barrier integrity. *J Biol Chem*. 2006; 281: 29190-200.

8. Kurreck J, Wyszko E, Gillen C and Erdmann VA. Design of antisense oligonucleotides stabilized by locked nucleic acids. *Nucleic acids research*. 2002; 30: 1911-8.

9. Small GR, Hadoke PW, Sharif I, et al. Preventing local regeneration of glucocorticoids by 11beta-hydroxysteroid dehydrogenase type 1 enhances angiogenesis. *Proceedings of the National Academy of Sciences of the United States of America*. 2005; 102: 12165-70.
